# Crystal structure of (2-{[(8-aminona­phthalen-1-yl)imino]­meth­yl}-4,6-di-*tert*-butyl­phenolato-κ^3^ *N*,*N*′,*O*)bromido­nickel(II)

**DOI:** 10.1107/S2056989018003651

**Published:** 2018-03-09

**Authors:** Patrick O’Brien, Matthias Zeller, Wei-Tsung Lee

**Affiliations:** a Department of Chemistry & Biochemistry, 1068 W. Sheridan Rd., Chicago, IL 60660, USA; b Department of Chemistry, Purdue University, 560 Oval Dr., W. Lafayette, IN 47907-2084, USA

**Keywords:** crystal structure, nickel complex, tridentate ligand, square-planar coordination

## Abstract

The title compound, [NiBr(C_25_H_29_N_2_O)], contains an Ni^II^ atom with a slightly distorted square-planar coordination environment defined by one O and two N atoms from the 2-{[(8-aminona­phthalen-1-yl)imino]­meth­yl}-4,6-di-*tert*-butyl­phenolate ligand and a bromide anion. The Ni—O and Ni—N bond lengths are slightly longer than those observed in the phenyl backbone counterpart, which can be attributed to the larger steric hindrance of the naphthyl group in the structure of the title compound. The mol­ecule as a whole is substanti­ally distorted, with both the planar naphthalene-1,8-di­amine and imino–meth­yl–phenolate substitutents rotated against the NiN_2_OBr plane by 38.92 (7) and 37.22 (8)°, respectively, giving the mol­ecule a twisted appearance. N—H⋯Br hydrogen bonds and N—H⋯C(π) contacts connect the mol­ecules into dimers, and additional C—H⋯Br contacts, C—H⋯π inter­actions, and an offset stacking inter­action between naphthyl units inter­connect these dimers into a three-dimensional network.

## Abstract 2

The coordination environment of the Ni^II^ atom is slightly distorted square planar, whereas the appearance of the whole mol­ecule is twisted.

## Chemical context   

There has been an emergent inter­est in the design and synthesis of non-symmetrical iminoaryl bis­(salen)-based ligands because of their facile synthesis and tunable properties. As a result, their nickel complexes have been used in a variety of applications and properties, including metal–organic frameworks (Crane & MacLachlan, 2012 ▸), catalysis for styrene polymerization (Ding *et al.*, 2017 ▸), unique redox behavior (Rotthaus *et al.*, 2006 ▸; Kochem *et al.*, 2013 ▸), and non-linear optics (Cisterna *et al.*, 2015 ▸; Trujillo *et al.*, 2010 ▸). One of the synthetic methods utilizes the half-unit Schiff base as a precursor for the preparation of non-symmetrical iminoaryl bis­(salen) ligands. Surprisingly, ligands are mostly limited to phenyl derivatives as the backbone. Some metal complexes bearing non-symmetrical iminona­phthyl bis­(salen) ligands have been reported in the literature (Villaverde *et al.*, 2011 ▸; Boghaei & Mohebi, 2002 ▸; Sundaravadivel *et al.*, 2013 ▸, 2014 ▸), but their crystal structures were not determined. As part of our work on the synthesis of nickel complexes bearing non-symmetrical iminoaryl bis­(salen)-based ligands, we report here the crystal structure of (2-{[(8-aminona­phthalen-1-yl)imino]­meth­yl}-4,6-di-*tert*-butyl­phenolato-κ^3^ *N*,*N*′,*O*)bromidonickel(II), (I).

## Structural commentary   

**Figure 1:**
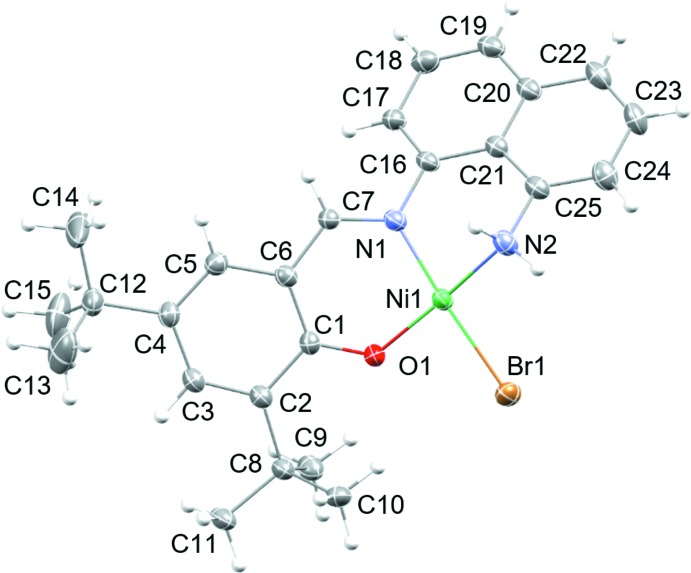
The mol­ecular structure of the title compound showing atom labels, with displacement ellipsoids at the 50% probability level.

The mol­ecular structure of the title compound, (I), is given in Fig. 1 ▸, with selected bond lengths and angles collated in Table 1 ▸. The structure confirms the nickel cation to be four-coordinate and bound by two N atoms (imine N1 and amine N2), the phenolic O atom (O1), and the Br atom (Br1). The amino nitro­gen atom (N2H_2_) is neutral, with both hydrogen atoms well-defined in difference electron density maps. The O1—C1 bond length of 1.312 (4) Å indicates a phenolate resonance form for the ligand. The Schiff base double N1=C7 bond is within the range expected for a metal-coordinating Schiff base–imine fragment.
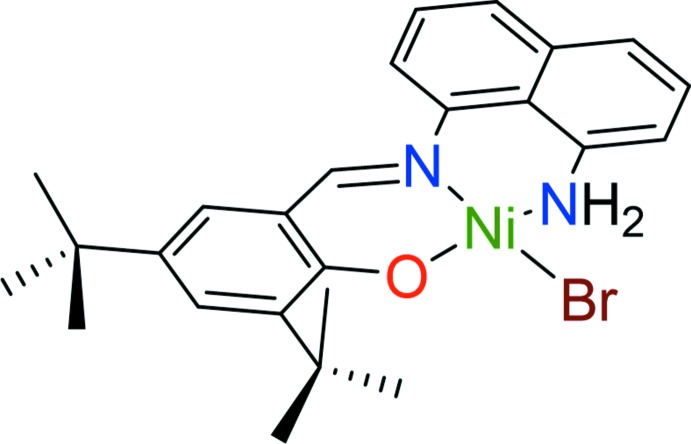


The coordination environment around the Ni^II^ cation can be best described as slightly distorted square-planar, with an r.m.s deviation from planarity for the NiN_2_OBr fragment of 0.0943 Å. Inter­estingly, the Ni1—N1, Ni1—N2, and Ni1—O1 bond lengths are slightly longer than those observed in the phenyl backbone counterpart of (I), [Ni(NNO)OAc] (II) (NNO = 2-{[(2-amino­phen­yl)imino]­meth­yl}-4,6-di-*tert*-butyl­phenolate; Ding *et al.*, 2017 ▸), which could be attributed to the increased steric bulk of the naphthyl backbone in (I). In line with this increased steric demand are the value for the angle N2—Ni1—O1 [170.15 (11)°], and that of the torsion angle C6—C7—N1—C16 [163.1 (3)°], which are significantly larger than those observed for (II) (176 and 178°, respectively). The steric profile of the aryl backbone appears to play an important role in altering both bond lengths and angles around the coordination center.

The increased steric demand in (I) does not substanti­ally affect the bond lengths and angles of the individual ligand fragments. Both the naphtyl as well as the imino­methyl phenolate fragments are essentially planar, with r.m.s deviations from planarity of only 0.062 and 0.072 Å, respectively (the least-squares planes include the N and O atoms attached to the fragments). They do, however, yield to the steric strain by substanti­ally rotating out of the plane of the NiN_2_OBr plane, and with respect to each other, giving the mol­ecule as a whole a twisted appearance. The dihedral angle of the naphthalene-1,8-di­amine unit with the central NiN_2_OBr plane is 38.92 (7)°, the equivalent angle of the imino­methyl phenolate substitutent is 37.22 (8)°. The inter­planar angle between the two organic fragments is 50.33 (5)°. This contrasts starkly with (II). The less sterically strained counterpart of (I) is essentially planar, with inter­planar angles of the NiN_2_O_2_ fragment with the phenyl­ene di-amine of only 5.91 and 7.39° [note that there are two independent mol­ecules in the structure of (II)], and of only 7.08 and 3.58° towards the imino­methyl phenolate fragments.

## Supra­molecular features   

**Figure 2:**
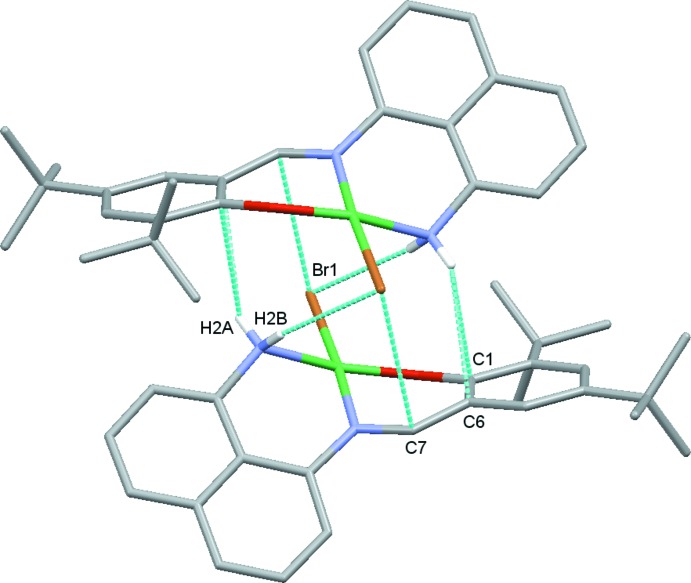
View of one of the dimers in (I), showing the N—H⋯Br hydrogen bonds and N—H⋯C(π) contacts. H atoms not involved in the inter­actions are omitted for clarity.

**Figure 3:**
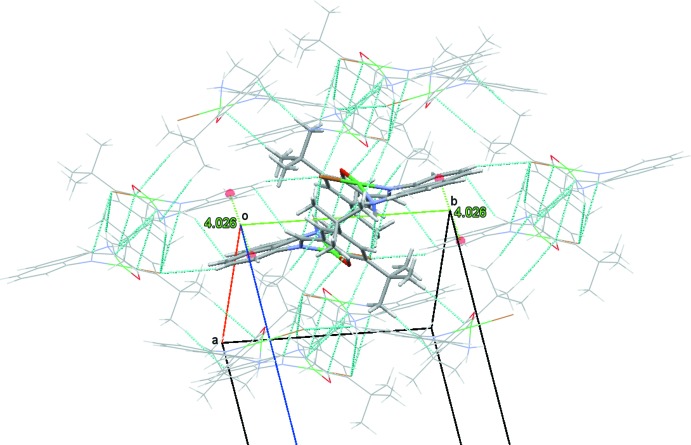
View of the inter­molecular inter­actions for (I), showing N—H⋯Br hydrogen bonds and N—H⋯C(π) contacts as well as C—H⋯Br contacts, C—H⋯π inter­actions, and the offset stacking inter­action between naphthyl units that inter­connects dimers into a three-dimensional framework. For clarity, only one central dimer is shown in stick mode, the surrounding mol­ecules in wireframe style.

The crystal-packing of (I) is steered by a number of medium strength and weak inter­molecular inter­actions. Most prominent is an inter­molecular N—H⋯Br hydrogen bond, Table 2 ▸, which connects individual mol­ecules into dimers. The hydrogen bond involves H2*B* of the amine group. The other amine H atom, H2*A*, does not form a hydrogen bond. Instead, it inter­acts with the π electron cloud of the phenolate ring, with two close N—H⋯C(π) contacts (Table 2 ▸). These latter inter­actions appear to provide additional synergy for the formation of the N—H⋯Br bridged dimers, Fig. 2 ▸. Other inter­molecular inter­actions in (I) are less directional. They involve a series of C—H⋯Br contacts, C—H⋯π inter­actions, and an offset stacking inter­action between naphthyl units of neighboring mol­ecules. Combined, these inter­actions connect the more tightly bound dimers into a three-dimensional network, Fig. 3 ▸.

## Database survey   

The most recent version of the Cambridge Structural Database (Version 5.39, updated November 2017; Groom *et al.*, 2016 ▸) has no entries related to iminona­phthyl mono(salen) supported metal complexes. However, a closely related compound, a nickel(II) complex bearing an imino­phenyl mono(salen) ligand, has been reported as its acetate complex, and has been compared to the title compound in the *Structural commentary*. A broader exploration showed eight entries corresponding to imino­phenyl mono(salen) ligands, including two aluminum (Muñoz-Hernández *et al.*, 2000 ▸), one copper (Ding *et al.*, 2014 ▸), two palladium (Vicente *et al.*, 1993 ▸, Liu *et al.*, 2010 ▸), one rhenium (Lane *et al.*, 2011 ▸), one ruthenium (Eltayeb *et al.*, 2007 ▸), and one tin (Yearwood *et al.*, 2002 ▸) complexes.

## Synthesis and crystallization   

Starting materials were commercially available and were used without further purification.

*Ligand synthesis*: 3,5-di-tertbutyl-2-hydro­benzaldehyde (1.00 g, 4.27 mmol) dissolved in ethanol (20 ml) was added to 1,8-di­aminona­phthalene (1.36 g, 8.53 mmol) in ethanol (20 ml) in a 100 ml round-bottom flask. The reaction mixture was refluxed for 24 h. Volatiles were removed under reduced pressure, and the residue was crystallized at 253 K to yield light-purple crystals (1.17 g, 73%). ^1^H NMR (300 MHz, C_6_D_6_, *d*): δ, 8.76 (*s*, 1H, C*H*), 7.63 (*d*, 1H, *J* = 2.1 Hz, Ar*H*), 7.26 (*d*, 2H, *J* = 8.1 Hz, Ar*H*), 7.18–7.13 (*m*, 2H, Ar*H*), 6.81 (*d*, 1H, *J* = 1.8 Hz, Ar*H*), 6.05 (*d*, 2H, *J* = 7.2 Hz, Ar*H*), 4.66 (*s*, 1H, O*H*), 3.72 (*s*, 2H, N*H_2_*), 1.71 [*s*, 9H, ArC(C*H*_3_)], 1.41 [*s*, 9H, ArC(C*H*_3_)].

*Synthesis of the title compound*: To a stirred solution of (*E*)-2-{[(8-aminona­phthalen-1-yl)imino]­meth­yl}-4,6-di-*tert*-butyl­phenol (80 mg, 0.21 mmol) in THF (3 mL) at ambient temperature under an N_2_ atmosphere was added a suspension of potassium *tert*-butoxide (26 mg, 0.24 mmol) in THF (2 mL) for 2 h. Solid NiBr_2_(DME) (69 mg, 0.22 mmol) was added, and the resulting slurry was stirred for 18 h at ambient temperature. Volatiles were removed under reduced pressure, and the residue was extracted with toluene and filtered through Celite. The filtrate was dried in *vacuo* to yield a dark-red solid (21 mg, 95%). Crystals suitable for X-ray diffraction were grown from a concentrated solution in Et_2_O at ambient temperature.

## Refinement   

Crystal data, data collection and structure refinement details are summarized in Table 3 ▸. H atoms attached to carbon atoms were positioned geometrically and constrained to ride on their parent atoms, with C—H bond lengths of 0.95 Å for alkene and aromatic moieties, and 0.98 Å for aliphatic CH_3_ moieties, respectively. Methyl H atoms were allowed to rotate but not to tip to best fit the experimental electron density. Amine H atom positions were refined with N—H distances restrained to 0.88 (2) Å. *U*_iso_(H) values were set to a multiple of *U*_eq_(C/N) with 1.5 for CH_3_, and 1.2 for C—H and N—H units, respectively. Reflections (0 0 2), (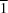 0 2) and (0 1 3) were obstructed by the beam stop and were omitted from the refinement.

## Supplementary Material

Crystal structure: contains datablock(s) I, global. DOI: 10.1107/S2056989018003651/wm5439sup1.cif

Structure factors: contains datablock(s) I. DOI: 10.1107/S2056989018003651/wm5439Isup2.hkl

CCDC reference: 1827007

Additional supporting information:  crystallographic information; 3D view; checkCIF report

## Figures and Tables

**Table 1:** Selected geometric parameters (Å, °)

N1—Ni1	1.880 (3)	Ni1—Br1	2.3330 (5)
N2—Ni1	1.922 (3)	C1—O1	1.312 (4)
O1—Ni1	1.850 (2)	C7—N1	1.305 (4)
			
O1—Ni1—N1	92.82 (10)	O1—Ni1—Br1	90.32 (7)
O1—Ni1—N2	170.15 (11)	N1—Ni1—Br1	176.24 (8)
N1—Ni1—N2	87.66 (12)	N2—Ni1—Br1	89.61 (9)
			
C6—C7—N1—C16	163.1 (3)		

**Table 2:** Hydrogen-bond geometry (Å, °)

*D*—H⋯*A*	*D*—H	H⋯*A*	*D*⋯*A*	*D*—H⋯*A*
N2—H2*B*⋯Br1^i^	0.88 (2)	2.98 (2)	3.827 (3)	162 (4)
N2—H2*A*⋯C1^i^	0.88 (2)	2.84 (4)	3.285 (4)	113 (3)
N2—H2*A*⋯C6^i^	0.88 (2)	2.90 (3)	3.589 (4)	137 (3)
C18—H18⋯Br1^ii^	0.95	2.93	3.624 (4)	131
C13—H13*A*⋯Br1^iii^	0.98	2.96	3.804 (6)	145
C11—H11*B*⋯C1^iv^	0.98	2.77	3.741 (5)	169
C9—H9*C*⋯C5^iv^	0.98	2.76	3.730 (5)	169
C7—H7⋯C19^v^	0.95	2.71	3.518 (5)	144

Symmetry codes: (i) 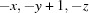; (ii) 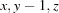; (iii) 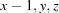; (iv) 
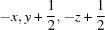
; (v) 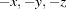.

**Table 3:** Experimental details

Crystal data	
Chemical formula	[NiBr(C_25_H_29_N_2_O)]
*M*_r_	512.12
Crystal system, space group	Monoclinic, *P*2_1_/*c*
Temperature (K)	100
*a*, *b*, *c* (Å)	9.7626 (3), 10.9008 (4), 22.0679 (7)
β (°)	98.0315 (14)
*V* (Å^3^)	2325.43 (13)
*Z*	4
Radiation type	Mo *K*α
μ (mm^−1^)	2.57
Crystal size (mm)	0.55 × 0.44 × 0.12

Data collection	
Diffractometer	Nonius KappaCCD
Absorption correction	Multi-scan (*SCALEPACK*; Otwinowski & Minor, 1997 ▸)
*T*_min_, *T*_max_	0.245, 0.735
No. of measured, independent and observed [*I* > 2σ(*I*)] reflections	11680, 5755, 4738
*R*_int_	0.045
(sin θ/λ)_max_ (Å^−1^)	0.705

Refinement	
*R*[*F* ^2^ > 2σ(*F* ^2^)], *wR*(*F* ^2^), *S*	0.046, 0.128, 1.07
No. of reflections	5755
No. of parameters	283
No. of restraints	2
H-atom treatment	H atoms treated by a mixture of independent and constrained refinement
Δρ_max_, Δρ_min_ (e Å^−3^)	1.07, −1.25

Computer programs: *COLLECT* (Nonius, 1998 ▸), *HKL-3000* (Otwinowski & Minor, 1997 ▸), *SHELXS97* (Sheldrick, 2008 ▸), *SHELXL2018* (Sheldrick, 2015 ▸) and *shelXle* (Hübschle *et al.*, 2011 ▸), *Mercury* (Macrae *et al.*, 2006 ▸) and *publCIF* (Westrip, 2010 ▸).

## supplementary crystallographic information

### Crystal data

**Table:**

[NiBr(C_25_H_29_N_2_O)]	*F*(000) = 1056
*M**_r_* = 512.12	*D*_x_ = 1.463 Mg m^−^^3^
Monoclinic, *P*2_1_/*c*	Mo *K*α radiation, λ = 0.71073 Å
*a* = 9.7626 (3) Å	Cell parameters from 11680 reflections
*b* = 10.9008 (4) Å	θ = 2.1–30.1°
*c* = 22.0679 (7) Å	µ = 2.57 mm^−^^1^
β = 98.0315 (14)°	*T* = 100 K
*V* = 2325.43 (13) Å^3^	Plate, black
*Z* = 4	0.55 × 0.44 × 0.12 mm

### Data collection

**Table:**

Nonius KappaCCD diffractometer	5755 independent reflections
Radiation source: fine focus X-ray tube	4738 reflections with *I* > 2σ(*I*)
Graphite monochromator	*R*_int_ = 0.045
ω and φ scans	θ_max_ = 30.1°, θ_min_ = 2.1°
Absorption correction: multi-scan (SCALEPACK; Otwinowski & Minor, 1997)	*h* = 0→13
*T*_min_ = 0.245, *T*_max_ = 0.735	*k* = 0→15
11680 measured reflections	*l* = −30→31

### Refinement

**Table:**

Refinement on *F*^2^	Primary atom site location: structure-invariant direct methods
Least-squares matrix: full	Secondary atom site location: difference Fourier map
*R*[*F*^2^ > 2σ(*F*^2^)] = 0.046	Hydrogen site location: mixed
*wR*(*F*^2^) = 0.128	H atoms treated by a mixture of independent and constrained refinement
*S* = 1.07	*w* = 1/[σ^2^(*F*_o_^2^) + (0.0631*P*)^2^ + 4.3983*P*] where *P* = (*F*_o_^2^ + 2*F*_c_^2^)/3
5755 reflections	(Δ/σ)_max_ < 0.001
283 parameters	Δρ_max_ = 1.07 e Å^−^^3^
2 restraints	Δρ_min_ = −1.25 e Å^−^^3^

### Special details

**Table:**

Geometry. All esds (except the esd in the dihedral angle between two l.s. planes) are estimated using the full covariance matrix. The cell esds are taken into account individually in the estimation of esds in distances, angles and torsion angles; correlations between esds in cell parameters are only used when they are defined by crystal symmetry. An approximate (isotropic) treatment of cell esds is used for estimating esds involving l.s. planes.

### Fractional atomic coordinates and isotropic or equivalent isotropic displacement parameters (Å^2^)

**Table:**

	*x*	*y*	*z*	*U*_iso_*/*U*_eq_	
C1	−0.0553 (3)	0.4478 (3)	0.14421 (13)	0.0207 (6)	
C2	−0.1176 (3)	0.5200 (3)	0.18765 (13)	0.0215 (6)	
C3	−0.2474 (3)	0.4849 (3)	0.19962 (14)	0.0231 (6)	
H3	−0.288647	0.532890	0.228138	0.028*	
C4	−0.3239 (3)	0.3836 (3)	0.17296 (14)	0.0230 (6)	
C5	−0.2624 (3)	0.3152 (3)	0.13236 (14)	0.0228 (6)	
H5	−0.310127	0.246173	0.113465	0.027*	
C6	−0.1295 (3)	0.3445 (3)	0.11769 (13)	0.0202 (6)	
C7	−0.0640 (3)	0.2563 (3)	0.08365 (13)	0.0208 (6)	
H7	−0.111370	0.180965	0.074598	0.025*	
C8	−0.0394 (3)	0.6284 (3)	0.22098 (14)	0.0230 (6)	
C9	0.0896 (3)	0.5782 (3)	0.26142 (16)	0.0295 (7)	
H9A	0.150747	0.538983	0.235565	0.044*	
H9B	0.061397	0.517857	0.290193	0.044*	
H9C	0.138719	0.645847	0.284265	0.044*	
C10	0.0052 (4)	0.7237 (3)	0.17568 (16)	0.0301 (7)	
H10A	0.064332	0.683952	0.149067	0.045*	
H10B	0.056620	0.790083	0.198582	0.045*	
H10C	−0.077039	0.757407	0.150692	0.045*	
C11	−0.1291 (4)	0.6966 (3)	0.26186 (15)	0.0281 (7)	
H11A	−0.213815	0.725748	0.236934	0.042*	
H11B	−0.077523	0.766708	0.281208	0.042*	
H11C	−0.153307	0.640822	0.293557	0.042*	
C12	−0.4634 (3)	0.3499 (4)	0.19290 (16)	0.0288 (7)	
C13	−0.5585 (5)	0.4609 (5)	0.1875 (3)	0.0651 (16)	
H13A	−0.573130	0.489421	0.144959	0.098*	
H13B	−0.516131	0.526707	0.214029	0.098*	
H13C	−0.647545	0.438227	0.199999	0.098*	
C14	−0.5341 (5)	0.2460 (6)	0.1546 (3)	0.075 (2)	
H14A	−0.550635	0.270893	0.111563	0.112*	
H14B	−0.622540	0.227022	0.168740	0.112*	
H14C	−0.474709	0.173182	0.158954	0.112*	
C15	−0.4382 (5)	0.3110 (5)	0.2604 (2)	0.0546 (13)	
H15A	−0.526967	0.293766	0.274528	0.082*	
H15B	−0.390898	0.377284	0.284997	0.082*	
H15C	−0.380507	0.237058	0.264654	0.082*	
C16	0.1225 (3)	0.1628 (3)	0.04555 (14)	0.0218 (6)	
C17	0.1062 (3)	0.0524 (3)	0.07443 (15)	0.0247 (6)	
H17	0.048093	0.048630	0.105510	0.030*	
C18	0.1736 (4)	−0.0540 (3)	0.05882 (16)	0.0283 (7)	
H18	0.158249	−0.129379	0.078375	0.034*	
C19	0.2612 (3)	−0.0504 (3)	0.01577 (15)	0.0277 (7)	
H19	0.306526	−0.123154	0.005685	0.033*	
C20	0.2850 (3)	0.0613 (3)	−0.01401 (14)	0.0246 (6)	
C21	0.2139 (3)	0.1693 (3)	0.00004 (13)	0.0220 (6)	
C22	0.3818 (4)	0.0675 (4)	−0.05633 (15)	0.0307 (8)	
H22	0.426691	−0.005268	−0.066781	0.037*	
C23	0.4111 (4)	0.1759 (4)	−0.08210 (16)	0.0322 (8)	
H23	0.479367	0.179134	−0.108810	0.039*	
C24	0.3404 (4)	0.2833 (4)	−0.06927 (15)	0.0310 (7)	
H24	0.360980	0.358951	−0.087370	0.037*	
C25	0.2425 (3)	0.2789 (3)	−0.03089 (14)	0.0238 (6)	
N1	0.0550 (3)	0.2692 (3)	0.06375 (11)	0.0217 (5)	
N2	0.1631 (3)	0.3879 (3)	−0.02205 (12)	0.0259 (6)	
H2A	0.205 (4)	0.450 (3)	−0.0365 (18)	0.031*	
H2B	0.076 (2)	0.376 (4)	−0.0368 (18)	0.031*	
O1	0.0677 (2)	0.4768 (2)	0.13117 (10)	0.0229 (4)	
Ni1	0.12898 (4)	0.42762 (4)	0.05935 (2)	0.02024 (11)	
Br1	0.23263 (4)	0.61856 (3)	0.05095 (2)	0.02840 (11)	

### Atomic displacement parameters (Å^2^)

**Table:**

	*U*^11^	*U*^22^	*U*^33^	*U*^12^	*U*^13^	*U*^23^
C1	0.0199 (13)	0.0223 (16)	0.0202 (13)	0.0005 (11)	0.0044 (10)	0.0015 (11)
C2	0.0218 (14)	0.0229 (17)	0.0196 (13)	0.0028 (11)	0.0026 (10)	−0.0005 (11)
C3	0.0237 (14)	0.0261 (17)	0.0200 (13)	0.0030 (12)	0.0047 (11)	−0.0005 (12)
C4	0.0185 (13)	0.0284 (18)	0.0221 (14)	0.0012 (12)	0.0032 (11)	0.0018 (12)
C5	0.0244 (15)	0.0221 (17)	0.0220 (13)	−0.0017 (12)	0.0035 (11)	0.0000 (11)
C6	0.0222 (14)	0.0196 (16)	0.0189 (13)	0.0023 (11)	0.0037 (10)	0.0006 (11)
C7	0.0228 (14)	0.0192 (16)	0.0208 (13)	−0.0014 (11)	0.0042 (10)	−0.0018 (11)
C8	0.0238 (14)	0.0224 (17)	0.0234 (14)	0.0001 (12)	0.0056 (11)	−0.0047 (11)
C9	0.0249 (16)	0.034 (2)	0.0285 (16)	0.0002 (13)	0.0018 (12)	−0.0083 (14)
C10	0.0367 (19)	0.0199 (18)	0.0345 (17)	−0.0037 (13)	0.0081 (14)	−0.0065 (13)
C11	0.0293 (16)	0.0271 (19)	0.0284 (15)	0.0013 (13)	0.0062 (12)	−0.0089 (13)
C12	0.0220 (15)	0.035 (2)	0.0311 (16)	−0.0027 (13)	0.0090 (12)	−0.0011 (14)
C13	0.026 (2)	0.060 (3)	0.112 (5)	0.010 (2)	0.021 (2)	0.019 (3)
C14	0.049 (3)	0.107 (5)	0.078 (4)	−0.048 (3)	0.040 (3)	−0.055 (3)
C15	0.038 (2)	0.081 (4)	0.047 (2)	−0.013 (2)	0.0134 (19)	0.019 (2)
C16	0.0240 (14)	0.0201 (16)	0.0216 (13)	0.0020 (11)	0.0040 (11)	−0.0047 (11)
C17	0.0294 (16)	0.0200 (17)	0.0257 (14)	−0.0015 (12)	0.0078 (12)	−0.0027 (12)
C18	0.0323 (17)	0.0193 (18)	0.0332 (17)	−0.0002 (13)	0.0038 (13)	−0.0007 (13)
C19	0.0280 (16)	0.0242 (18)	0.0303 (16)	0.0030 (13)	0.0027 (12)	−0.0071 (13)
C20	0.0230 (14)	0.0266 (18)	0.0236 (14)	0.0031 (12)	0.0017 (11)	−0.0053 (12)
C21	0.0221 (14)	0.0228 (17)	0.0209 (13)	0.0022 (12)	0.0019 (11)	−0.0023 (11)
C22	0.0285 (17)	0.038 (2)	0.0263 (15)	0.0092 (14)	0.0057 (13)	−0.0048 (14)
C23	0.0296 (17)	0.043 (2)	0.0264 (15)	0.0091 (15)	0.0109 (13)	0.0027 (14)
C24	0.0334 (18)	0.036 (2)	0.0242 (15)	0.0051 (14)	0.0075 (13)	0.0040 (14)
C25	0.0254 (15)	0.0248 (18)	0.0209 (13)	0.0040 (12)	0.0023 (11)	−0.0026 (12)
N1	0.0243 (13)	0.0207 (14)	0.0206 (11)	0.0014 (10)	0.0051 (9)	−0.0025 (10)
N2	0.0311 (15)	0.0260 (16)	0.0218 (12)	0.0034 (11)	0.0081 (11)	0.0010 (11)
O1	0.0234 (11)	0.0219 (12)	0.0251 (10)	−0.0017 (9)	0.0086 (8)	−0.0029 (9)
Ni1	0.0229 (2)	0.0184 (2)	0.02046 (19)	0.00083 (14)	0.00663 (14)	−0.00010 (14)
Br1	0.03690 (19)	0.0209 (2)	0.02905 (17)	−0.00314 (13)	0.01042 (13)	0.00202 (12)

### Geometric parameters (Å, º)

**Table:**

N1—Ni1	1.880 (3)	C12—C13	1.520 (6)
N2—Ni1	1.922 (3)	C12—C14	1.520 (6)
N2—H2A	0.878 (19)	C12—C15	1.535 (5)
N2—H2B	0.878 (19)	C13—H13A	0.9800
O1—Ni1	1.850 (2)	C13—H13B	0.9800
Ni1—Br1	2.3330 (5)	C13—H13C	0.9800
C1—O1	1.312 (4)	C14—H14A	0.9800
C1—C6	1.420 (4)	C14—H14B	0.9800
C1—C2	1.439 (4)	C14—H14C	0.9800
C2—C3	1.384 (4)	C15—H15A	0.9800
C2—C8	1.537 (4)	C15—H15B	0.9800
C3—C4	1.414 (5)	C15—H15C	0.9800
C3—H3	0.9500	C16—C17	1.381 (5)
C4—C5	1.367 (4)	C16—N1	1.420 (4)
C4—C12	1.533 (4)	C16—C21	1.435 (4)
C5—C6	1.418 (4)	C17—C18	1.400 (5)
C5—H5	0.9500	C17—H17	0.9500
C6—C7	1.426 (4)	C18—C19	1.365 (5)
C7—N1	1.305 (4)	C18—H18	0.9500
C7—H7	0.9500	C19—C20	1.418 (5)
C8—C11	1.534 (4)	C19—H19	0.9500
C8—C9	1.538 (5)	C20—C22	1.420 (5)
C8—C10	1.546 (5)	C20—C21	1.423 (5)
C9—H9A	0.9800	C21—C25	1.422 (5)
C9—H9B	0.9800	C22—C23	1.359 (6)
C9—H9C	0.9800	C22—H22	0.9500
C10—H10A	0.9800	C23—C24	1.408 (5)
C10—H10B	0.9800	C23—H23	0.9500
C10—H10C	0.9800	C24—C25	1.364 (5)
C11—H11A	0.9800	C24—H24	0.9500
C11—H11B	0.9800	C25—N2	1.447 (4)
C11—H11C	0.9800		
			
O1—C1—C6	122.0 (3)	C12—C14—H14B	109.5
O1—C1—C2	120.0 (3)	H14A—C14—H14B	109.5
C6—C1—C2	118.0 (3)	C12—C14—H14C	109.5
C3—C2—C1	117.4 (3)	H14A—C14—H14C	109.5
C3—C2—C8	121.8 (3)	H14B—C14—H14C	109.5
C1—C2—C8	120.8 (3)	C12—C15—H15A	109.5
C2—C3—C4	125.5 (3)	C12—C15—H15B	109.5
C2—C3—H3	117.3	H15A—C15—H15B	109.5
C4—C3—H3	117.3	C12—C15—H15C	109.5
C5—C4—C3	116.4 (3)	H15A—C15—H15C	109.5
C5—C4—C12	123.1 (3)	H15B—C15—H15C	109.5
C3—C4—C12	120.4 (3)	C17—C16—N1	119.5 (3)
C4—C5—C6	121.8 (3)	C17—C16—C21	119.3 (3)
C4—C5—H5	119.1	N1—C16—C21	121.1 (3)
C6—C5—H5	119.1	C16—C17—C18	121.3 (3)
C5—C6—C1	121.0 (3)	C16—C17—H17	119.4
C5—C6—C7	117.4 (3)	C18—C17—H17	119.4
C1—C6—C7	120.8 (3)	C19—C18—C17	120.6 (3)
N1—C7—C6	126.2 (3)	C19—C18—H18	119.7
N1—C7—H7	116.9	C17—C18—H18	119.7
C6—C7—H7	116.9	C18—C19—C20	120.4 (3)
C11—C8—C2	111.6 (3)	C18—C19—H19	119.8
C11—C8—C9	108.6 (3)	C20—C19—H19	119.8
C2—C8—C9	108.4 (3)	C19—C20—C22	120.9 (3)
C11—C8—C10	106.9 (3)	C19—C20—C21	119.6 (3)
C2—C8—C10	111.9 (3)	C22—C20—C21	119.6 (3)
C9—C8—C10	109.4 (3)	C25—C21—C20	117.1 (3)
C8—C9—H9A	109.5	C25—C21—C16	124.1 (3)
C8—C9—H9B	109.5	C20—C21—C16	118.7 (3)
H9A—C9—H9B	109.5	C23—C22—C20	121.0 (3)
C8—C9—H9C	109.5	C23—C22—H22	119.5
H9A—C9—H9C	109.5	C20—C22—H22	119.5
H9B—C9—H9C	109.5	C22—C23—C24	120.1 (3)
C8—C10—H10A	109.5	C22—C23—H23	120.0
C8—C10—H10B	109.5	C24—C23—H23	120.0
H10A—C10—H10B	109.5	C25—C24—C23	120.1 (3)
C8—C10—H10C	109.5	C25—C24—H24	120.0
H10A—C10—H10C	109.5	C23—C24—H24	120.0
H10B—C10—H10C	109.5	C24—C25—C21	122.0 (3)
C8—C11—H11A	109.5	C24—C25—N2	119.3 (3)
C8—C11—H11B	109.5	C21—C25—N2	118.7 (3)
H11A—C11—H11B	109.5	C7—N1—C16	118.5 (3)
C8—C11—H11C	109.5	C7—N1—Ni1	118.9 (2)
H11A—C11—H11C	109.5	C16—N1—Ni1	122.6 (2)
H11B—C11—H11C	109.5	C25—N2—Ni1	118.5 (2)
C13—C12—C14	108.9 (4)	C25—N2—H2A	108 (3)
C13—C12—C4	110.2 (3)	Ni1—N2—H2A	108 (3)
C14—C12—C4	111.8 (3)	C25—N2—H2B	110 (3)
C13—C12—C15	108.1 (4)	Ni1—N2—H2B	95 (3)
C14—C12—C15	109.4 (4)	H2A—N2—H2B	117 (4)
C4—C12—C15	108.4 (3)	C1—O1—Ni1	122.3 (2)
C12—C13—H13A	109.5	O1—Ni1—N1	92.82 (10)
C12—C13—H13B	109.5	O1—Ni1—N2	170.15 (11)
H13A—C13—H13B	109.5	N1—Ni1—N2	87.66 (12)
C12—C13—H13C	109.5	O1—Ni1—Br1	90.32 (7)
H13A—C13—H13C	109.5	N1—Ni1—Br1	176.24 (8)
H13B—C13—H13C	109.5	N2—Ni1—Br1	89.61 (9)
C12—C14—H14A	109.5		
			
O1—C1—C2—C3	−179.9 (3)	C18—C19—C20—C21	−1.7 (5)
C6—C1—C2—C3	−1.3 (4)	C19—C20—C21—C25	179.6 (3)
O1—C1—C2—C8	−2.3 (4)	C22—C20—C21—C25	1.4 (4)
C6—C1—C2—C8	176.3 (3)	C19—C20—C21—C16	1.7 (4)
C1—C2—C3—C4	0.3 (5)	C22—C20—C21—C16	−176.4 (3)
C8—C2—C3—C4	−177.3 (3)	C17—C16—C21—C25	−177.6 (3)
C2—C3—C4—C5	0.4 (5)	N1—C16—C21—C25	−1.2 (5)
C2—C3—C4—C12	176.4 (3)	C17—C16—C21—C20	0.1 (4)
C3—C4—C5—C6	−0.2 (5)	N1—C16—C21—C20	176.5 (3)
C12—C4—C5—C6	−176.1 (3)	C19—C20—C22—C23	−175.9 (3)
C4—C5—C6—C1	−0.8 (5)	C21—C20—C22—C23	2.2 (5)
C4—C5—C6—C7	169.1 (3)	C20—C22—C23—C24	−3.0 (5)
O1—C1—C6—C5	−179.8 (3)	C22—C23—C24—C25	0.1 (5)
C2—C1—C6—C5	1.6 (4)	C23—C24—C25—C21	3.7 (5)
O1—C1—C6—C7	10.6 (5)	C23—C24—C25—N2	−175.1 (3)
C2—C1—C6—C7	−168.0 (3)	C20—C21—C25—C24	−4.4 (5)
C5—C6—C7—N1	176.8 (3)	C16—C21—C25—C24	173.3 (3)
C1—C6—C7—N1	−13.2 (5)	C20—C21—C25—N2	174.4 (3)
C3—C2—C8—C11	−6.1 (4)	C16—C21—C25—N2	−7.9 (5)
C1—C2—C8—C11	176.4 (3)	C6—C7—N1—C16	163.1 (3)
C3—C2—C8—C9	113.5 (3)	C6—C7—N1—Ni1	−17.6 (4)
C1—C2—C8—C9	−64.0 (4)	C17—C16—N1—C7	−31.8 (4)
C3—C2—C8—C10	−125.7 (3)	C21—C16—N1—C7	151.9 (3)
C1—C2—C8—C10	56.7 (4)	C17—C16—N1—Ni1	149.0 (2)
C5—C4—C12—C13	−130.8 (4)	C21—C16—N1—Ni1	−27.3 (4)
C3—C4—C12—C13	53.5 (5)	C24—C25—N2—Ni1	−137.7 (3)
C5—C4—C12—C14	−9.5 (5)	C21—C25—N2—Ni1	43.4 (4)
C3—C4—C12—C14	174.8 (4)	C6—C1—O1—Ni1	23.3 (4)
C5—C4—C12—C15	111.1 (4)	C2—C1—O1—Ni1	−158.1 (2)
C3—C4—C12—C15	−64.6 (4)	C1—O1—Ni1—N1	−41.9 (2)
N1—C16—C17—C18	−178.5 (3)	C1—O1—Ni1—Br1	140.2 (2)
C21—C16—C17—C18	−2.1 (5)	C7—N1—Ni1—O1	37.9 (2)
C16—C17—C18—C19	2.1 (5)	C16—N1—Ni1—O1	−142.9 (2)
C17—C18—C19—C20	−0.2 (5)	C7—N1—Ni1—N2	−132.3 (2)
C18—C19—C20—C22	176.4 (3)	C16—N1—Ni1—N2	46.9 (2)

### Hydrogen-bond geometry (Å, º)

**Table:**

*D*—H···*A*	*D*—H	H···*A*	*D*···*A*	*D*—H···*A*
N2—H2*B*···Br1^i^	0.88 (2)	2.98 (2)	3.827 (3)	162 (4)
N2—H2*A*···C1^i^	0.88 (2)	2.84 (4)	3.285 (4)	113 (3)
N2—H2*A*···C6^i^	0.88 (2)	2.90 (3)	3.589 (4)	137 (3)
C18—H18···Br1^ii^	0.95	2.93	3.624 (4)	131
C13—H13*A*···Br1^iii^	0.98	2.96	3.804 (6)	145
C11—H11*B*···C1^iv^	0.98	2.77	3.741 (5)	169
C9—H9*C*···C5^iv^	0.98	2.76	3.730 (5)	169
C7—H7···C19^v^	0.95	2.71	3.518 (5)	144

Symmetry codes: (i) −*x*, −*y*+1, −*z*; (ii) *x*, *y*−1, *z*; (iii) *x*−1, *y*, *z*; (iv) −*x*, *y*+1/2, −*z*+1/2; (v) −*x*, −*y*, −*z*.
